# Hormesis does not make sense except in the light of TOR-driven aging

**DOI:** 10.18632/aging.100411

**Published:** 2011-12-12

**Authors:** Mikhail V. Blagosklonny

**Affiliations:** Department of Cell Stress Biology, Roswell Park Cancer Institute, BLSC, L3-312, Buffalo, NY, 14263, USA

**Keywords:** Hormesis, aging, senescence, rapamycin, mTOR, damage, diseases

## Abstract

Weak stresses (including weak oxidative stress, cytostatic agents, heat shock, hypoxia, calorie restriction) may extend lifespan. Known as hormesis, this is the most controversial notion in gerontology. For one, it is believed that aging is caused by accumulation of molecular damage. If so, hormetic stresses (by causing damage) must shorten lifespan. To solve the paradox, it was suggested that, by activating repair, hormetic stresses eventually decrease damage. Similarly, Baron Munchausen escaped from a swamp by pulling himself up by his own hair. Instead, I discuss that aging is not caused by accumulation of molecular damage. Although molecular damage accumulates, organisms do not live long enough to age from this accumulation. Instead, aging is driven by overactivated signal-transduction pathways including the TOR (Target of Rapamycin) pathway. A diverse group of hormetic conditions can be divided into two groups. “Hormesis A” inhibits the TOR pathway. “Hormesis B” increases aging-tolerance, defined as the ability to survive catastrophic complications of aging. Hormesis A includes calorie restriction, resveratrol, rapamycin, p53-inducing agents and, in part, physical exercise, heat shock and hypoxia. Hormesis B includes ischemic preconditioning and, in part, physical exercise, heat shock, hypoxia and medical interventions.

Paraphrasing the famous quote “Nothing in Biology Makes Sense Except in the Light of Evolution”, one can say that nothing in aging makes sense except in the light of TOR-driven quasi-programmed aging, a continuation of developmental growth driven by growth-promoting pathways. And life span extension by mild damage makes no sense, if aging is a decline caused by accumulation of damage.

## Conventional view on aging

**Figure 1:**
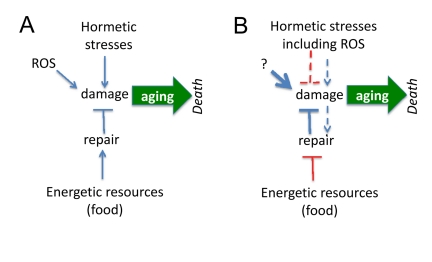
Paradoxical links between damage and aging (**A**) If agings is caused by damage, then hormetic damage should accelerate aging. Also food by providing resources should becelerate aging. Both prediction contradict observations, making the model incorrect. (**B**) Paradoxical model assumes that (a) damage decrease damage and (b) the less resources (food), the more resources can be used for anti-aging repair. These assumptions are paradoxical but nevertheless are needed to fit predictions and observations. Paradoxical links are shown in red.

It is believed that aging is a decline, deterioration due to accumulation of random molecular and cellular damage caused by free radicals, radiation, stresses, pathogens, toxins, carcinogens, mistakes in replication/translation, protein misfolding and even mechanical forces. If aging is caused by damage, then damaging stresses would accelerate aging (Figure 1A). However, mild stresses (including oxidative stress) can extend life span in different species [1-30].

How this can be reconciled with the conventional theory of aging. There are 3 options:

First, hormesis is an artefact. Certainly there are many artefacts in this field. Yet, there are also solid data especially on the life-extension by calorie restriction, ROS, heat shock and phytochemicals.

Second, the phenomenon of hormesis rules out the conventional theory of aging. Furthermore, as it was already reviewed, “damage-induced aging” theory was ruled out by other evidence too [31-38]. It was discussed that aging is not the life-long accumulation of molecular damage, is not decline and is not caused by reactive oxygen species (ROS) [35].

Third, instead of rejecting the damage-induced theory, paradoxical assumptions were suggested to reconcile it with hormesis (Figure 1B). To explain extension of lifespan by mild and repeated stresses, it was suggested that (a) mild stresses stimulate maintenance and repair pathways and (b) cause adaptation of cells and the ability to tolerate stronger stresses. Let us briefly review the attempt to reconcile hormesis and molecular damage-driven aging.

## Conventional explanations of hormesis

### Heat shock

Repeated exposure to mild heat shock increases life span in *Drosophila* [11]. It was suggested that mild heat shock reduces damage and protein aggregation by activating internal antioxidant, repair and degradation processes [2, 3]. In other words, chronic cellular stress may prolong life span by either activating repair mechanisms or by causing cell adaptation or both [1-3].

### Calorie restriction

It was suggested that calorie restriction (CR) is a low-intensity stressor, which enhances the ability of animals to cope with intense stressors [4]. For example, in young rodents, CR causes an increase in the afternoon peak concentration of plasma corticosterone, a stress hormone [5, 39]. Another explanation is completely paradoxical. If damage is not completely repaired because resources for repair are limited by food supply, as suggested by Kirkwood [40], this predicts that CR (less resources) must accelerate aging (Figure 1A). It was also suggested by Kirkwood that, although the repair is limited by insufficient resources, the more resources (food), the less repair (Figure 1B). The reason of self-contradiction is that aging, according to the same theory, is purposefully regulated and the organism may choose to age slower [40]. The paradoxes of this point of view were recently discussed [34, 38, 41] and will not be discussed here further.

### ROS

In some studies, an increased production of reactive oxygen species (ROS) correlated with extended life span in different species. To explain such paradoxical results it was suggested that an increased ROS in turn increases resistance to ROS, thus extending life span [1, 12, 14]. It was suggested that ROS leads to a condition of mild stress, which in turn enhances vitality [12]. In *C. elegans* reduced glucose availability promoted formation of ROS, induces catalase activity, and increased oxidative stress resistance, cumulating in extension of life span [15].

## Two noticeable problems

First, it is paradoxical to decrease damage by causing damage (Figure 1B). There is no similar example in medicine. If one wishes to prevent stroke due to high blood pressure, one needs to decrease blood pressure not to increase it. Examples are endless including weight control to prevent diabetes and quitting smoking to prevent lung cancer. No one will advocate “mild and repeated” smoking to prevent lung cancer even though it might activate the defenses. The simple reason is that DNA damage is *actually* involved in cancer initiation. But even cancer-promoting damage is not random: mutations activate growth-promoting pathways including PI3K/mTOR, the most universal alteration in cancer [42-45]. And cancer-initiating damage does not cause cellular decline (in contrast, cancer cells are very robust and hyper-functional), is not sufficient to cause cancer, requiring rounds of cell replication, selection [46, 47] and organismal aging [48]. By slowing down aging, CR and rapamycin delay cancer (without affecting mutations). The notion that aging promotes cancer is beyond the topic of this article and cannot be discussed here. The point here is that since DNA damage contributes to cancer, no one will suggest hormetic smoking or radioactivity (at any doses) to delay cancer. In analogy, if damaging hormesis may delay aging, aging cannot be possibly be caused by damage.

Second problem is the suggestion that mild hormetic stresses protect against severe stresses. What are these severe stresses? Even according to the conventional view, aging is not caused by accidental injures that are stronger than hormetic damage. It is caused by ‘everyday’ ROS and other background stresses. Let's ask a straightforward question. Are hormetic stresses stronger or weaker than those that cause aging? And there is no answer. If damage that drives aging does not sufficiently induce protective response, then hormesis is stronger than aging-causing stresses. Then why is the purpose of hormesis to protect from stronger stresses? This question will be easily answered in the light of TOR-driven aging.

## Solution: a new view on aging

If aging is driven by damage, then damage must accelerate aging. If hormesis induces damage and slows down aging, then aging is not driven by damage. So a straightforward explanation is that aging is not caused by accumulation of molecular damage [36]. It was predicted “that five years from now, current opponents will take the TOR-centric model for granted, which then will become new dogma (ironically)” [35].

It is becoming evident that ROS do not cause aging and furthermore often is associated with longevity [26, 27, 30, 49, 50-66].

While rejecting ROS-driven aging, scientists still do not dare to reject the view that aging is a decline due to accumulation of random damage. Yet data exclude not only ROS but also damage as a cause of aging. For example, in *C elegans*, CR did not decrease the accumulation of spontaneous mutations with age but nevertheless extended life span [54].

Yes, perhaps, molecular damage accumulates but organisms do not live long enough to age from this accumulation. Even humans, long-living organisms, do not die from a decline due to such an accumulation. (And of course worm that lives just 5 days [67] cannot possibly accumulate deadly levels of molecular damage). Instead any human being has died from age-related diseases, which are caused by active cellular processes initiated by hyper-activation of signaling pathways including mTOR [36, 68]. The pathogenesis of diseases is well known. In contrast, a mysterious cellular decline due to accumulation of molecular damage is the fiction of gerontology, unknown in medical science.

Instead of trying to adopt the phenomenon of hormesis to the view on aging as accumulation of random molecular damage, we will reconsider the view on aging.

## Aging: TOR-driven process

**Figure 2:**
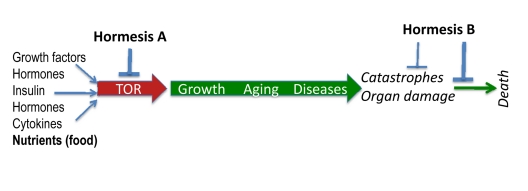
TOR-centric model of aging Nutrients (food), growth factors, cytokines, insulin and hormones activate the nutrient-sensing TOR (Target of Rapamycin) pathway, which promotes growth and then aging, causing age-related diseases. In turn, diseases cause non-random organ damage and death. Hormesis type A inhibits TOR thus slowing down aging. Hormesis type B increases aging-tolerance and tolerance to complications of age-related diseases.

The nutrient-sensing TOR pathway is activated by insulin, growth factors and nutrients (Figure 2). In turn, it increases protein synthesis, stimulates ribosomal biogenesis and cell mass growth (causing cell hypertrophy), inhibits autophagy, induces accumulation of aggregation-prone proteins, increases growth factors (GF) secretion and causes resistance to GF and insulin [69-79].

The TOR pathway drives cellular mass growth. In proliferating cells, cellular growth is balanced by cell division [80]. In quiescent (resting) cells, growth-promoting pathways drive senescence [80, 81]. When the cell cycle is blocked but mTOR is still active, it causes hypertrophic, hyperactive, hyper-functional (for example, hyper-secretory) phenotype, with compensatory resistance to signals such as insulin and growth factors [82, 83] and compensatory lysosomal activation. In other words, mTOR converted quiescence into senescence, the process that was named gerogenic conversion or geroconversion or gerogenesis [83].

In brief, TOR causes cellular hyper-functions, specific to each tissue such as bone resorption by osteoclasts and arterial wall tonus by smooth muscle cells, manifested as osteoporosis and hypertension, for instance. This eventually damages organs (aging-induced catastrophes). As discussed, secretory phenotype [84] or (more generally) hyper-functional phenotype [31] links cell senescence to organismal aging and specifically to age-related diseases. The TOR pathway is involved in diseases such as cancers, type II diabetes and its complications (retinopathy and renal hypertrophy), age-related macular degeneration, obesity, atherosclerosis, cardiac hypertrophy, organ fibrosis (liver, renal and cardiac fibrosis), osteoporosis, Alzheimer's and Parkinson's diseases and arthritis [31, 32, 38, 68, 74, 85-89]. Organisms die from age-related diseases. TOR is involved in all of them [31, 68]. In other words, TOR limits life span by accelerating age-related diseases. In humans (and other mammals), age-related diseases are manifestations of aging that *actually* limit life span.

Age-related diseases culminate in sudden catastrophes (Figure 2). For example, death of cardiocytes, during myocardial infarction, is often caused ischemia due to the arterial occlusion. Such occlusions result from increased coagulation and platelet hyper-function, atherosclerosis, inflammatory state and high blood pressure. Age-related osteoporosis culminates in the broken hip, diabetes – in renal failure, hypertension – in stroke, just to name a few. Inhibition of the TOR pathway prolongs life span in yeast, worm, flies and mice [77, 90-107]. Genes for aging (named gerogenes [37]) constitute the TOR pathway [37]. Genes for longevity (named gerosuppressors) antagonize the TOR pathway [37]. Furthermore, some “anti-aging” hormetic agents antagonize this pathway too.

## Longevity: (a) slow aging and (b) aging-tolerance

Life span can be extended by either (a) slowing down aging and (b) by increasing aging tolerance, defined as the ability to survive complications (catastrophes) of aging [36].

a. Obviously, inhibition of aging should extend life span and delay age-related diseases. For example, calorie restriction (CR) slows aging. CR delays age-related diseases such as cancer and atherosclerosis, thus extending life span. In other words, inhibition of mTOR-driven aging delays catastrophic complications of aging: namely, complications of age-related diseases such as stroke, myocardial infarction (Figure 2). These non-random catastrophes actually cause death.

b. But inhibition of aging (and delaying diseases) is not the only way to extend life span. The second way is to increase aging tolerance, which allows an organism to survive catastrophes caused by age-related diseases.

### Why organisms age

The existence of aging is well understood from the evolutionary perspective and was discussed in detail. Roughly speaking, in the wild, organisms do not live long enough to experience aging. Therefore forces of natural selection against aging are weak. Only in protected environment (humans, domestic and laboratory animals) die from aging.

### Why organisms have low aging-tolerance

From the evolutionary perspective, organisms do not tolerate aging for the same reason why they age in the first place. In the wild, organisms do not live long enough to experience aging and therefore organisms are not naturally shaped to experience complications of aging. Organisms are not selected by nature for aging-tolerance. For example, parts of myocardium depend on a single coronary artery. The occlusion of a coronary artery causes life-threatening ischemia. Collateral arteries would prevent ischemia. Natural selection would favor such anatomical re-design, if it will extend reproductive life span. If humans were routinely reproducing after the age of 70, then variations with additional branches of coronary arteries would be selected.

Thus myocardial ischemia, due to artery occlusion is one of the most common causes of death. The occlusion may result from thrombosis of atherosclerotic coronary arteries. But if the ischemic zone would receive blood supply from a collaterally artery, the organism would survive the catastrophe. Thus, anatomical modifications of a myocardial blood supply would increase aging-tolerance without affecting aging itself. Noteworthy, this is how coronary stents extend lifespan without affecting aging. Most medical treatments increase aging tolerance, thus extending an average life span despite chronic age-related diseases. In contrast, pharmacological suppression of aging would increase healthy lifespan by postponing diseases [108].

## Two types of hormesis: (a) slowing down aging and (b) increasing aging-tolerance

Hormetic stresses include two groups of agents that (a) slow down aging by inhibiting the TOR pathway and (b) increase aging tolerance, without affecting the aging process (Figure 2). We will call them hormesis A and hormesis B. Examples of hormesis A are calorie restricttion, rapamycin, resveratrol and p53-inducing agents. Examples of hormesis B are adaptive preconditioning to ischemia and coronary bypass. Heat shock, hypoxia and physical exercise belong to both groups.

## Hormesis A

### Calorie restriction

Caloric restriction (CR) markedly extends life span in diverse species from yeast to mammals and delays the occurrence and/or slows progression of age-associated diseases [109-118]. It was suggested that CR slows down aging via the TOR pathway in yeast, *C. elegans* and *Drosophila* and mammals [34, 93, 94, 119-[122]. In humans, it has been shown that nutrients activate TOR in the muscle tissue, causing insulin-resistance, preventable by rapamycin [123]. Starvation or CR deactivates the TOR pathway [71, 96, 97, 124]. Thus, by inhibiting TOR, CR may slow down aging and extend lifespan.

## Chemical hormesis

Plants, microorganisms and sea animals produce toxic agents that inhibit or damage microtubules, DNA and many other vital targets. Due to their toxicity, some of them are used as anti-cancer drugs, although nature did not created them for that purpose. Nature of course created these poisons to hurt predators and competitors [125, 126]. Similarly, rapamycin is an antifungal antibiotic produced by bacteria. TOR stimulates growth in response to nutrients. Therefore, soil bacteria produce rapamycin to inhibit yeast growth. While inhibiting TOR-dependent growth, rapamycin slows down TOR-dependent aging in older yeast [93, 94]. Given that cancer (like aging) is “a form of growth”, the mTOR pathway is activated in cancer. And, although not created for that purpose by nature, inhibitors of mTOR are used as anticancer agents [42, 43, 127, 128]. I wish to emphasize again that bacteria produce rapamycin neither as a medicine for longevity nor as an anticancer drug, but as an antifungal antibiotic. Simply the same signaling pathways that are involved in growth also are involved in cancer and aging [88]. Growth suppressants may suppress aging because aging is a continuation of growth, driven by the same TOR/S6K pathway [129]. To extend lifespan, they either should inhibit the TOR pathway or increase aging tolerance (Figure 2).

### Rapamycin

Rapamycin extends life span in yeast, drosophila and mice [94, 98, 99, 130, 101, 103, 130]. It is indicated for almost all age-related diseases [31, 48, 68, 131]. Rapamycin is not toxic for normal cells at concentrations that exceed therapeutic levels 1000 fold [132], [133]. There are no side effects of high dose rapamycin in healthy volunteers [123, 134]. Rapamycin has been used in children [135] and in pregnant women [136]. Despite common misconception that rapamycin is an immunosuppressant, rapamycin improves immunity in mice when used appropriately [137-139]. As an anti-aging modality, rapamycin could be used in doses and schedules that do not cause side effects [132].

### Resveratrol

Resveratrol, a natural agent found in grape skins, prevents age-related diseases and extends lifespan in several species [10, 140-145], including mice on high-fat diet [144]. Resveratrol activates sirtuins [10, 146-147], which inhibit the TOR pathway (see for references [148]). Resveratrol indirectly antagonizes the mTOR/S6K pathway upstream and downstream [129, 149-153], in part via activation of AMPK and sirtuins [154-160]. Plants produce resveratrol to protect grapes from parasites. But, coincidentally, inhibition of the TOR pathway slows down aging. Thus, the anti-aging effect of resveratrol may be just a side effect of targeting mTOR. Yet, at concentrations that inhibit mTOR the gerosuppressive effect of resveratrol is limited by its toxicity [149]. This is not surprising, given that resveratrol inhibits mTOR at micro-molar concentrations at which it also inhibits multiple unrelated targets. This may explain why anti-aging effects in mice may be limited by resveratrol toxicity [161]. As a potential solution, resveratrol at sub-therapeutic doses could be combined with rapamycin.

### Metformin

The anti-diabetic drug metformin activates AMPK, which in turn antagonizes the mTOR pathway [24, 162-165]. Metformin decreases insulin resistance, prevents diabetes and its complications, decrease incidence of heart diseases and cancer [166-168]. In rodents, metformin prolongs life span or prevents cancer or both [169-174].

### Physical exercise

As an example of hormesis A, chronic increase in physical activity inhibits mTOR/S6K1 in rat skeletal muscle [175]. Physical activity can also increase aging-tolerance, acting as hormesis B.

### Heat shock

The TOR pathway stimulates Cap-dependent protein synthesis. Elevated temperature inhibits cap-dependent protein synthesis. Thus, heat shock blocks TOR-stimulated protein synthesis. For example, heat shock protein Hsp27 inhibits translation during heat shock by binding eIF4G [176]. Therefore, heat shock acts as “hormesis A” by imitating TOR inhibition. The small heat-shock proteins also delay the onset of polyglutamine-expansion protein aggregation, suggesting that these proteins couple the normal aging process to this type of age-related disease [177]. Also, HSPs and chaperones can increase resistance or tolerance to catastrophic complications of aging, defining them also as hormesis B.

### Hypoxia

Depending on conditions, HIF-1 and hypoxia have different effects on longevity [30, 65, 178]. mTOR via phosphorylation of S6K/S6 and 4EBP1 induce cap-dependent translation. In contrast, hypoxia decreases cap-dependent translation. Hypoxia inhibits protein synthesis by deactivation of the mTOR pathway as well as by inactivation of eIF2α and eEF2 factors [179-186].

## p53-inducing stresses

DNA damage induces p53, which is known to inhibit mTOR pathway both upstream and downstream of mTOR [187-197]. Induction of p53 by nutlin-3a can suppress senescent phenotype or suppress conversion of quiescence into senescence [197-200]. The gero-suppressive effect is evident only when p53 is capable to inhibit mTOR [198, 201]. In certain conditions, p53 may act as an anti-aging agent [202-207].

## Hormesis B

Hormesis B extends life span by increasing aging tolerance. Mild stresses prepare organism to catastrophes caused by diseases of aging. Examples of catastrophes include stroke and myocardial ischemia. The occlusion of a cerebral artery for 60 min (injurious ischemia) damages the brain. The occlusion of the same cerebral artery for 15 min (preconditioning) protects from the damage caused by injurious ischemia [208]. Similarly, severe myocardial ischemia causes irreversible injury. Mild ischemia protects the heart from severe ischemia. Similarly, by inducing HSPs, heat shock may protect the myocardium from severe ischemia. Repeated, transient ischemic episodes or heat shock augment the ischemic tolerance of affected myocardium. Upregulation of immediate early genes and heat shock genes plays an important role in myocardial adaptation to acute ischemic stress [209]. Also, hormetic stresses can cause growth of collateral arteries. This coronary collateral function can preserve ischemic myocardium [210].

The cardioprotection against myocardial injury by regular exercise may include the development of collateral coronary arteries and induction of myocardial heat shock proteins [211].

Similarly, coronary bypass protected heart from ischemia. Although we do not call such medical procedures hormesis, there is no strict borderline between them and hormesis B. For example, reconditioning, hypoxia, and stresses may “train” cardiomyocutes to survive acute episode of coronary thrombosis. Also, it develops small blood vessels that could compensate for the occlusion of main artery.

Now we can solve the second problem of hormesis (see “two noticeable problems”). The answer is: hormetic stresses protect from stronger stresses. But these stronger stresses are not those that cause aging (aging is not caused by any stresses). These are complications of aging or age-related diseases. We call them catastrophes. Hormesis B protects from lethal catastrophes.

## Conclusion

The hypothesis that aging is NOT driven by accumulation of random damage allows us to explain hormesis. Type A hormesis antagonizes the TOR pathway (Figure 2). Hormesis B causes stresses including damaging stresses. Since aging is not caused by damage, this does not contribute to aging but instead may cause aging-tolerance, thus protecting organisms from lethal consequences of aging-induced catastrophes.
